# Environmental Variables and Eyrie Site Characteristics Associated with Breeding Productivity of the Barbary Falcon (*Falco peregrinus pelegrinoides*): Potential Implications for Release Site Evaluation

**DOI:** 10.3390/life16091482

**Published:** 2026-09-05

**Authors:** Monif AlRashidi, Mohammed Shobrak

**Affiliations:** 1 Department of Biology, Faculty of Science, University of Ha’il, Ha’il 55476, Saudi Arabia; 2 National Centre for Falcons, Riyadh 13535, Saudi Arabia; 3 National Centre for Wildlife, Prince Saud Al-Faisal for Wildlife Research, Taif 26618, Saudi Arabia; shobrak@saudibirds.org

**Keywords:** *Falco peregrinus pelegrinoides*, breeding productivity, eyrie cliff aspect, conservation translocation, ERA5-Land, release site planning, wind exposure

## Abstract

Environmental variables and eyrie site characteristics may influence reproductive output, yet their associations with Barbary falcon (*Falco peregrinus pelegrinoides*) productivity remain poorly understood in the Arabian Peninsula. We quantified fledgling production at 59 monitored eyries in northwestern and southwestern Saudi Arabia during the 2026 breeding season. Poisson generalized linear models evaluated February–May climatic conditions, pair type, eyrie cliff–wind alignment (CosDiff), and elevation and eyrie height where available. Monthly analyses for February–April and COM–Poisson sensitivity models assessed temporal consistency and robustness to departures from Poisson equidispersion. Mean productivity was 1.97 fledglings per eyrie (SD = 1.25; range = 0–4). In the best ranked additive model, a one standard deviation increase in seasonal mean wind speed (0.678 m s^−1^) was associated with 19.5% lower expected productivity (IRR = 0.805, 95% CI = 0.660–0.982, *p* = 0.032), and more direct eyrie cliff alignment with incoming prevailing wind was associated with 18.7% lower expected productivity (IRR = 0.813, 95% CI = 0.677–0.976, *p* = 0.026). Monthly wind estimates were comparable across February–April. These findings may inform preliminary release site evaluation, but multi-year studies with cliff-scale measurements are needed to determine whether these associations persist across breeding seasons.

## 1. Introduction

Breeding productivity in cliff-nesting raptors can reflect the combined influence of atmospheric conditions and breeding site characteristics [1,2,3]. Adverse weather may affect reproductive output by increasing the thermoregulatory and energetic demands placed on incubating and brooding adults and by increasing the mortality risk of embryos and nestlings [2,4,5]. Cliff structure and the position, degree of shelter, and orientation of the eyrie may determine the extent to which adults, eggs, and nestlings are exposed to wind, precipitation, and temperature extremes [2,3].

In the Peregrine falcon (*Falco peregrinus*), cold and wet weather has been associated with reduced breeding success, although the magnitude of these effects can depend partly on nest site shelter and orientation relative to prevailing weather [2]. Heavy rainfall directly increased nestling mortality in Arctic Canada, whereas experimentally sheltering nests from rainfall improved nestling survival [5]. A long-term study in South Greenland found that lower Peregrine falcon breeding success was associated with both the frequency of exceptionally cold days and a composite measure integrating extreme temperature and precipitation events [6]. Wind may additionally increase eyrie exposure by driving precipitation into otherwise sheltered sites [2].

Evidence from other cliff-nesting raptors further demonstrates the relevance of breeding site characteristics. In western Alaska, greater exposure at Gyrfalcon (*Falco rusticolus*) nesting sites was associated with increased adult nest attendance and reduced productivity [3]. In southeastern Spain, Bonelli’s eagles (*Aquila fasciata*) selected taller nesting cliffs located on steeper slopes than were generally available [7].

The Barbary falcon (*Falco peregrinus pelegrinoides*) is distributed from the Canary Islands across North Africa and the Middle East to parts of Central Asia [8,9]. It is distinguished from other peregrine forms by its relatively small body size, pale plumage, and characteristic rufous nape patches [10,11]. Its taxonomic status has been debated, and it has been treated as the full species *Falco pelegrinoides* in some taxonomic and regional conservation treatments [8,12]. However, genetic evidence indicates limited differentiation from other Peregrine falcon populations, and the Barbary falcon is generally treated as the subspecies *F. p. pelegrinoides* [11,13].

The capture of adults and collection of nestlings for falconry have been identified as important conservation pressures in Saudi Arabia, where the Barbary falcon ranked fourth among the falcon taxa used in falconry [14]. At the Arabian Peninsula scale, Symes et al. [12] estimated a population of approximately 2600 mature individuals and inferred a decline of about 30% over three generations, resulting in its classification as regionally vulnerable. In their Saudi Arabian species account, Boland and Alsuhaibany [15] reported approximately 600 breeding pairs and classified the Barbary falcon as regionally endangered despite describing its population trend as stable or possibly increasing, because the population within Arabia remained small.

Although previous studies have examined aspects of Barbary falcon ecology across Saudi Arabia, including conservation threats, breeding territory occupancy and productivity, the occurrence of unpaired adults [14,16,17], the associations of climatic conditions, topography, and eyrie site characteristics with variation in fledgling productivity remain poorly understood. Therefore, the primary objective of this study was to determine whether fledgling productivity among monitored Barbary falcon eyries in northwestern and southwestern Saudi Arabia was associated with climatic conditions, topography, and eyrie site characteristics, and to evaluate the potential relevance of these associations to future release site evaluation.

## 2. Materials and Methods

### 2.1. Study Area

**Figure 1:**
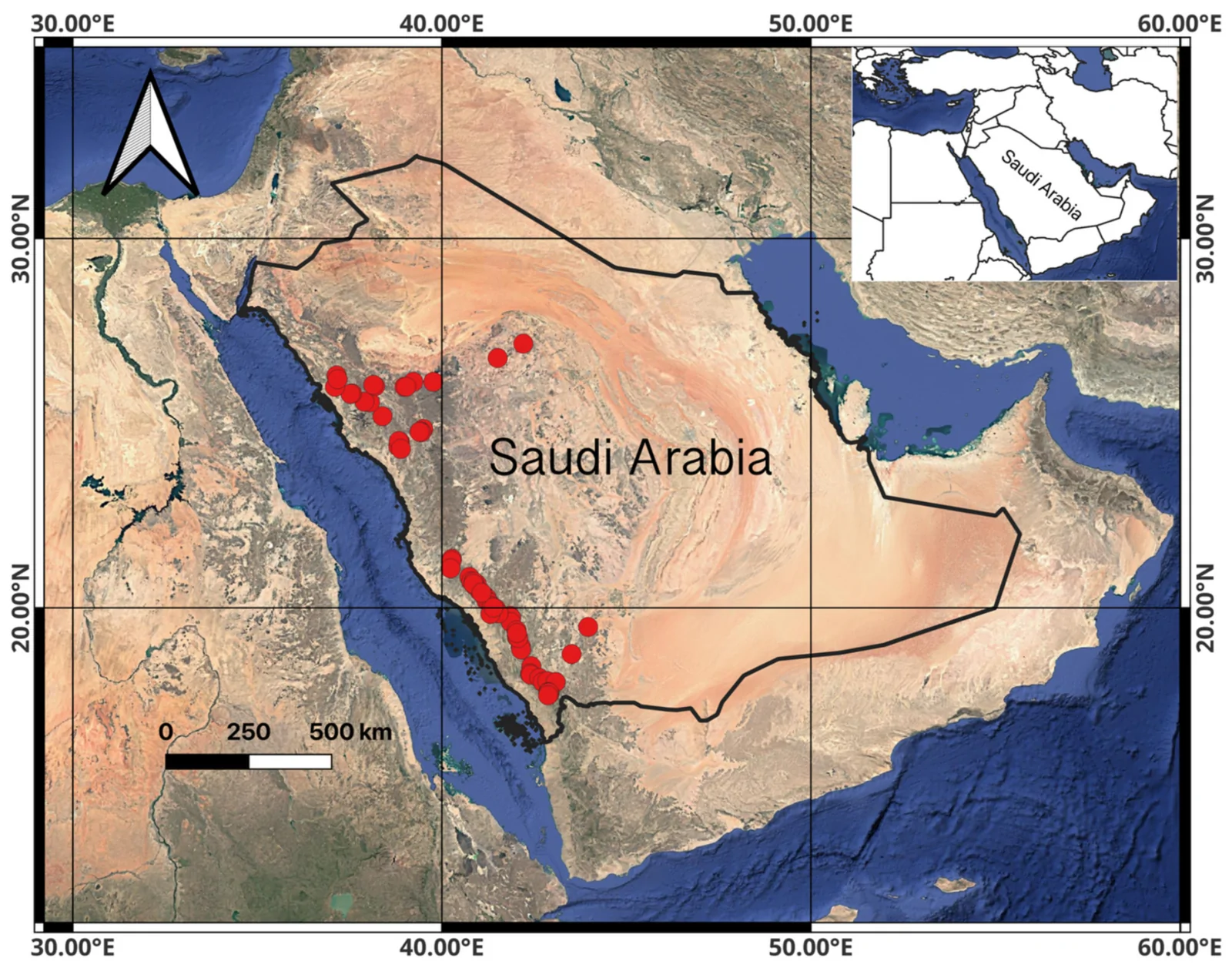
Distribution of Barbary falcon eyries included in this study in northwestern and southwestern Saudi Arabia. Red circles indicate monitored eyrie locations.

This study included known Barbary falcon (*Falco peregrinus pelegrinoides*) breeding territories in two broad spatial clusters in northwestern and southwestern Saudi Arabia, encompassing mountainous landscapes with steep cliffs, rocky outcrops, and deep valleys (Figure 1). The map was prepared in QGIS v. 3.40 [18].

### 2.2. Field Surveys and Eyrie Measurements

Field surveys were conducted from 23 February to 30 June 2026, spanning egg laying and incubation through fledging. Fieldwork was designed to visit all known breeding eyries. Access was constrained at some remote mountainous eyries by travel time and rugged terrain. In some cases, reaching one eyrie required an entire day of fieldwork, involving travel by four-wheel drive vehicles followed by prolonged travel on foot.

To distinguish the monitored breeding site from the physical nest, the term *eyrie* was used for a monitored breeding site associated with a known breeding cliff, whereas *nest* referred to the specific physical location used for egg laying, incubation, and rearing nestlings. Accordingly, *eyrie cliff aspect* denoted the orientation of the cliff supporting the eyrie, whereas *nest entrance aspect* denoted the orientation of the nest entrance.

Ground observations were combined with surveys using a DJI Air 3S unmanned aerial vehicle (SZ DJI Technology Co., Ltd., Shenzhen, China) (Aircraft Registration Mark: KSA-292932). Where the nest entrance had been identified, the UAV was flown to within approximately 5–10 m to obtain high-resolution photographs and videos, with each flight restricted to less than 5 min per eyrie. Some incubating adults remained at the nest even when the UAV approached to approximately 5 m. Adults that temporarily left the nest generally returned within 5 min after the UAV withdrew. As a precautionary measure, the aircraft was fitted with high-visibility red and yellow adhesive tape to increase its visibility to breeding adults and reduce the risk of accidental collision. Adults occasionally approached the UAV during surveys, but no attacks or collisions were observed.

The elevation above sea level at the UAV take-off location was recorded using a handheld Garmin eTrex GPS receiver (Garmin International, Inc., Olathe, KS, USA). Because the DJI Air 3S displayed vertical altitude relative to its take-off point rather than elevation above sea level, the absolute elevation of each eyrie was estimated by adding the signed UAV altitude displayed when the aircraft reached the eyrie to the GPS-recorded elevation of the UAV take-off location. The UAV automatically recorded the geographic coordinates associated with each photograph, which were used to determine the coordinates of each eyrie. The horizontal UAV distance was not incorporated into the elevation calculation.

The eyrie height on the cliff was measured using a Nohawk laser rangefinder (Model HK-600P; Tianjin Lookout Photoelectric Technology Co., Ltd., Tianjin, China). Where direct access to the area immediately below the cliff was feasible, the height was measured from that point. When reaching the area immediately below the cliff would have required excessive time, the nearest accessible point below the eyrie was identified, and the vertical heights of both the reference point and the *eyrie* were measured using the rangefinder. The difference between these two measurements was used as the eyrie height. Thus, this variable represents the vertical separation between the eyrie and the accessible reference point below it rather than the total height of the cliff. These measurements were selected because cliff geometry, elevation, and shelter have been associated with occupancy or productivity in Peregrine falcons and with habitat use by cliff-nesting raptor communities [19,20].

At each formal visit, the number of adults detected—neither, one, or both—and evidence of breeding activity were recorded. Failure to detect adults during an individual visit was not considered evidence that the eyrie was unoccupied; occupancy and breeding status were determined from observations accumulated across visits, including territorial activity, prey deliveries, nestlings, and recently fledged young. Nest contents and structural characteristics were recorded only when directly confirmed. Confirmation of clutch size was particularly difficult because females remained at the nest for prolonged periods during incubation and many nest entrances could not be identified reliably from the ground. Exact nest locations were sometimes identified following prey delivery events during incubation or through the increased frequency of provisioning visits after hatching.

### 2.3. Monitoring Effort and Determination of Fledgling Productivity

Each eyrie was subjected to at least two formal field visits during the breeding season, with some eyries visited on three occasions. The intervals between formal visits were not standardized because of differences in accessibility and travel requirements. In addition, a local monitor from the Hadad Program of the National Centre for Falcons was assigned to each eyrie and generally monitored the resident pair from a distance at weekly intervals until the young fledged. The final number of fledglings was recorded during the last formal field visit and cross-checked against the local monitor’s weekly observations.

Breeding productivity was defined as the number of young confirmed to have fledged from each monitored eyrie. A young falcon was classified as a fledgling once it had left the nest and was observed outside it, either perched on the occupied cliff or undertaking its initial flights. Where the precise nest entrance had not been located, fledglings observed on or immediately around the occupied cliff were assigned to the corresponding eyrie only when their location and association with the resident pair supported that assignment, including parental attendance, prey delivery, or defensive behaviour.

To avoid repeated counting, productivity was based on the maximum number of dependent young observed simultaneously during direct observation or within a single photographic or video sequence; observations made on different dates were not summed. A productivity value of zero was assigned only when the presence of a pair at the eyrie had been confirmed and repeated monitoring through the expected fledging period produced no evidence that young had fledged. Failure to locate the precise nest entrance alone was not treated as evidence of zero productivity.

### 2.4. Analytical Sample

The field database contained 68 monitored eyries. Six eyries at which only a male was confirmed and three eyries at which the presence of a pair could not be confirmed across the monitoring period were excluded from the productivity analysis. The analytical sample therefore comprised 59 eyries at which both members of a pair were confirmed. The eyrie was treated as the analytical unit, and breeding productivity was determined according to the monitoring and classification criteria described above.

### 2.5. Directly Confirmed Nest Attributes

Nest form, nest substrate or lining, clutch size, and nest entrance aspect were summarized descriptively only for the 15 nests in which eggs and structural characteristics were directly confirmed. Nest entrance aspect was recorded in degrees and assigned to one of eight compass sectors for descriptive reporting. These variables were not included as predictors of productivity because comparable direct observations were unavailable for the remaining eyries and the resulting category sizes were insufficient for reliable inferential modelling.

### 2.6. Environmental Variables

Meteorological variables were derived from the ERA5-Land Daily Aggregated reanalysis produced by the European Centre for Medium-Range Weather Forecasts, which provides globally consistent land surface fields at an approximate spatial resolution of 9 km [21]. Daily values were extracted at the coordinates of each eyrie in Google Earth Engine [22] using a scale parameter of 11,132 m. For each day, 10 m wind speed was calculated as √(u^2^ + v^2^) from the eastward (u) and northward (v) wind components. Mean wind speed for February–May 2026 was then calculated as the arithmetic mean of these daily speed magnitudes. Seasonal prevailing wind direction was calculated by first averaging u and v across the same period and then converting the resultant vector to the meteorological direction from which the wind originated; daily bearings were not averaged arithmetically.

Six continuous seasonal climate variables were screened: mean and maximum wind speed, cumulative precipitation, and minimum, mean, and maximum air temperature. Pairwise Pearson correlations were inspected, and variables with |r| ≥ 0.70 within the same climatic domain were considered redundant. The productivity models retained mean wind speed, cumulative precipitation, and minimum air temperature; maximum wind speed and mean and maximum temperature were excluded from modelling after screening. Rainfall, temperature, and weather exposure were considered because they have previously been associated with Peregrine falcon breeding outcomes [2,5,6].

### 2.7. Eyrie Cliff Aspect and Directional Alignment

For all 59 analytical eyries, the aspect of the cliff supporting each eyrie was measured to the nearest degree using a compass application. The observer stood directly above or below the eyrie and oriented the compass in the same outward-facing direction as the cliff face supporting the eyrie. For analysis, the measured bearings were assigned to eight sectors (N, NE, E, SE, S, SW, W, and NW), and each sector was represented by its midpoint bearing (0°, 45°, 90°, 135°, 180°, 225°, 270°, and 315°, respectively). The eyrie cliff aspect was distinguished from the directly measured nest entrance aspect, which was available for 15 nests.

The continuous eyrie cliff–wind alignment index was calculated from the midpoint bearing of the cliff aspect sector and the local seasonal prevailing wind bearing as CosDiff = cos(θcliff − θwind). This sector midpoint representation introduces a maximum angular quantization error of ±22.5°. CosDiff approaches +1 when the cliff faces toward the direction from which the prevailing wind arrives, 0 when the cliff face is approximately perpendicular to the incoming wind, and −1 when the cliff faces away from the incoming wind. This index quantifies directional alignment only and does not directly measure airflow, turbulence, or shelter conditions within the eyrie.

### 2.8. Statistical Analysis

All analyses were conducted in R 4.4.1 [23]. Continuous predictors were standardized to mean zero and unit standard deviation before modelling, so exponentiated coefficients are incidence rate ratios (IRRs) for a one standard deviation increase. Pair type, seasonal mean wind speed, cumulative precipitation, minimum air temperature, and CosDiff were available for all 59 eyries. Pair type was classified as naturally formed (n = 44) or release-supported (n = 15). A release-supported pair comprised a resident unpaired male associated with an established eyrie and a female released through the Hadad Program to facilitate pair formation. The smaller release-supported group limited the power of the pair-type comparison, so a non-significant result was not interpreted as evidence of equivalence. Elevation and eyrie height were available for 39 eyries and were evaluated separately rather than reducing the primary sample.

Fledgling productivity was analysed with generalized linear models with a Poisson error distribution and log link [24]. After the correlation screening described above, an exploratory, biologically structured candidate model set was defined to represent distinct ecological domains and selected plausible interactions; it was not preregistered and was not generated by fitting every possible predictor combination. Models were ranked using Akaike’s information criterion corrected for small sample size (AICc) [25], with ΔAICc ≤ 2 indicating similar support. The same structures were fitted with the mean parameterized Conway–Maxwell–Poisson distribution [26] using glmmTMB [27] to assess robustness to departures from Poisson equidispersion. Generalized variance inflation factors were examined before coefficient interpretation [28]. No mixed-effects model was fitted because each eyrie contributed one productivity value from one breeding season.

Nested likelihood ratio tests evaluated whether adding alignment to the wind-only model and whether adding each interaction improved fit beyond its constituent main effects. Model adequacy was assessed using DHARMa simulation-based residual diagnostics with 5000 simulations [29]. Because the eyries were distributed in two broad spatial clusters, residual spatial autocorrelation in the focal Poisson model was evaluated using Moran’s I calculated from DHARMa scaled residuals. Spatial weights were defined as the inverse of pairwise Haversine distances between eyrie coordinates and were row-standardized; significance was assessed using a two-sided test [30]. Potentially influential observations were assessed using Cook’s distance, leverage, standardized Pearson residuals, and R influence diagnostics [31], followed by leave-one-eyrie-out refitting of the focal seasonal models. These diagnostics were used to evaluate model robustness and were not used to exclude observations automatically.

Topographic sensitivity models were fitted using Poisson errors for the 39 eyries with complete measurements of elevation and eyrie height. Before interpreting this subset, wind speed and fledgling productivity were compared between the 39 eyries with and the 20 eyries without complete topographic measurements. To avoid collinearity, precipitation and elevation were not included together. Wind-only, wind-plus-elevation, wind-plus-eyrie height, and wind-plus-both formulations were compared by AICc and nested likelihood ratio tests.

Cliff sector counts were compared with the equal frequency expectation using a chi-square goodness-of-fit test to determine whether the frequencies across the eight sectors were globally non-uniform. Adjusted standardized residuals were then used only to identify which sectors contributed most to the global statistic, and their *p* values were Holm-corrected to control the family-wise probability of false positive sector claims [32]. This analysis describes non-uniform use but cannot demonstrate directional selection because the availability of suitable cliff faces in each sector was not measured [33]. Exact prevailing wind bearings were analysed with the circular package [34]. The circular mean and mean resultant length summarized the dominant bearing and its concentration, while Rayleigh’s test evaluated whether the wind bearings showed a unimodal departure from circular uniformity [35]. For relative eyrie cliff/wind angles, Rayleigh’s test addressed unimodal concentration, whereas Kuiper’s omnibus test addressed any departure from circular uniformity, including non-unimodal patterns; the tests were therefore complementary rather than duplicates. The Kuiper *p* value was estimated from 99,999 Monte Carlo simulations because the package output supplied critical value decisions rather than the required continuous *p* value. A three-class goodness-of-fit test assessed the facing, oblique, and opposite categories against null probabilities determined by their respective angular widths. Exact one-sided binomial tests separately evaluated whether broadly oblique aspects exceeded their 75% angular expectation and whether a narrower band centred on 45° exceeded its 25% expectation. Because these five relative angle tests addressed related formulations of the same biological question, their *p* values were Holm-corrected as one family; no single unadjusted result was treated as sufficient evidence of directional preference.

### 2.9. Monthly Temporal Sensitivity Analysis

Meteorological predictors were analysed separately for February, March, and April, corresponding broadly to the principal egg-laying and early incubation, hatching, and early nestling periods, respectively. For each month, monthly mean wind speed, total precipitation, and minimum air temperature were standardized and evaluated using the same seven null, single-predictor, additive, wind by precipitation, and wind by minimum air temperature formulations. The two interactions were fitted separately to avoid estimating both simultaneously in the 59-eyrie sample.

Monthly candidate models were fitted to all 59 eyries under both Poisson and COM–Poisson distributions and ranked by AICc. Nested likelihood ratio tests evaluated the two interactions separately in each month, and the six resulting Poisson *p* values were adjusted jointly by Holm’s method. These analyses were used to determine whether the seasonal wind–productivity association was driven primarily by conditions in a particular month or showed a similar direction and magnitude across February, March, and April.

## 3. Results

### 3.1. Analytical Sample and Breeding Productivity

**Figure 2:**
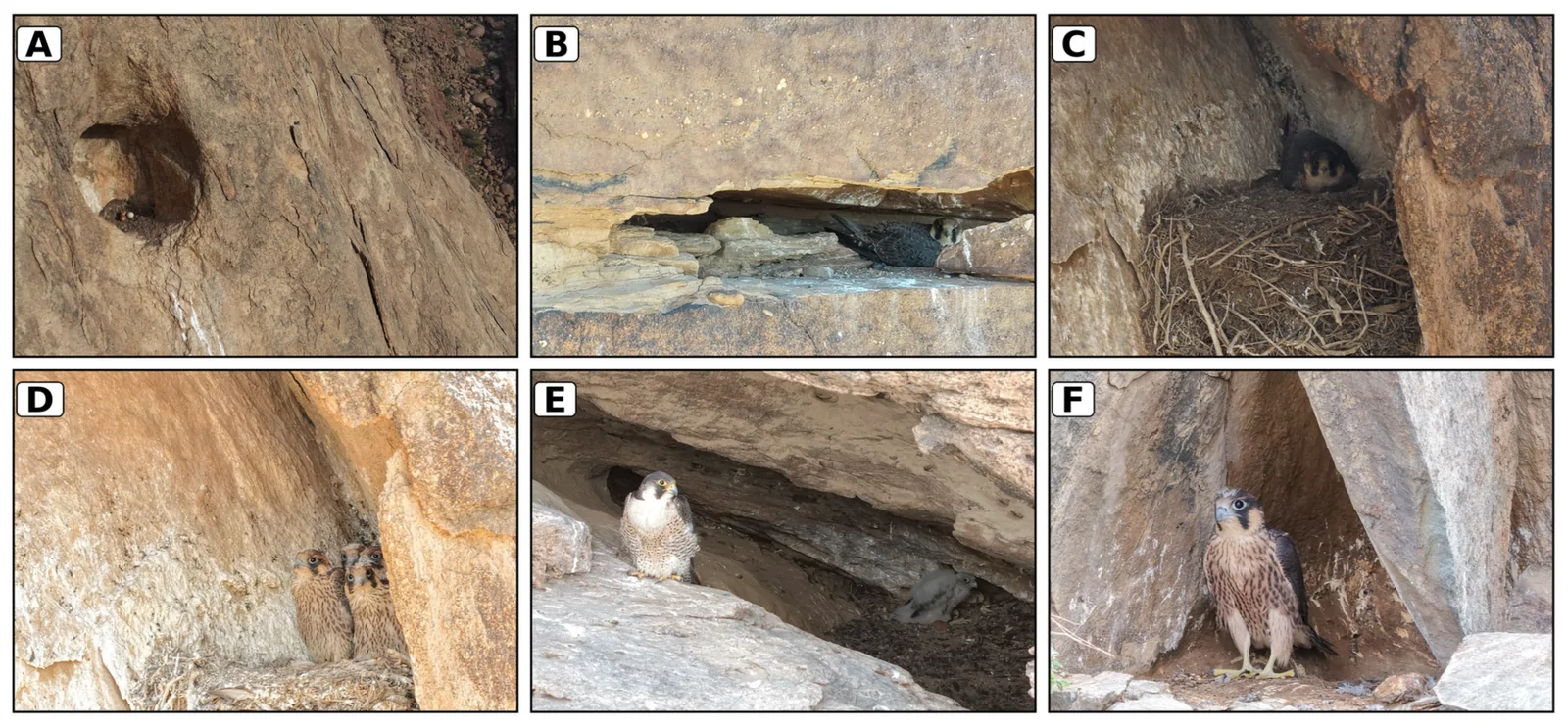
Representative directly confirmed Barbary falcon nests and breeding contents documented by UAV: (**A**) a rock cavity nest containing three eggs; (**B**) an adult incubating eggs in a rock crack nest; (**C**) confirmed use of an old raptor stick nest by an incubating adult; (**D**) four nestlings subsequently recorded in the same old raptor nest shown in panel C; (**E**) an adult female with one nestling and one unhatched egg in a rock cavity nest with a sandy floor and no visible added lining; and (**F**) a fully feathered nestling at the nest entrance shortly before fledging.

The primary analytical sample comprised 59 monitored eyries occupied by pairs. Mean productivity was 1.97 ± 1.25 fledglings per eyrie (median = 2; range = 0–4). Eleven eyries produced no fledglings (18.6%), nine produced one (15.3%), 14 produced two (23.7%), 21 produced three (35.6%), and four produced four (6.8%). Of the 59 breeding pairs, 44 were classified as naturally formed pairs, whereas 15 were classified as release-supported pairs. Seasonal environmental and topographic summaries are provided in Table 1, directly confirmed nest attributes for the 15 nests containing eggs are presented in Table 2, and representative examples of directly confirmed nests and their breeding contents are shown in Figure 2.

Categorical descriptors: 44 naturally formed and 15 release-supported pair types. Fledgling counts were 0 (n = 11), 1 (n = 9), 2 (n = 14), 3 (n = 21), and 4 (n = 4).

### 3.2. Candidate Model Comparison and Seasonal Wind Effect

The Poisson and COM–Poisson core global models had similar AICc values (Table S2). Variance inflation factors for the complete data predictors ranged from 1.13 to 3.24, indicating no severe collinearity after screening.

The additive mean wind plus eyrie cliff–wind alignment model ranked first under Poisson regression (M6; Table 3). The wind by alignment interaction model (M7) was the only other model within the predefined ΔAICc ≤ 2 criterion (Table 3). Models containing only mean wind or eyrie cliff–wind alignment received less support than M6 (Table 3).

**Figure 3:**
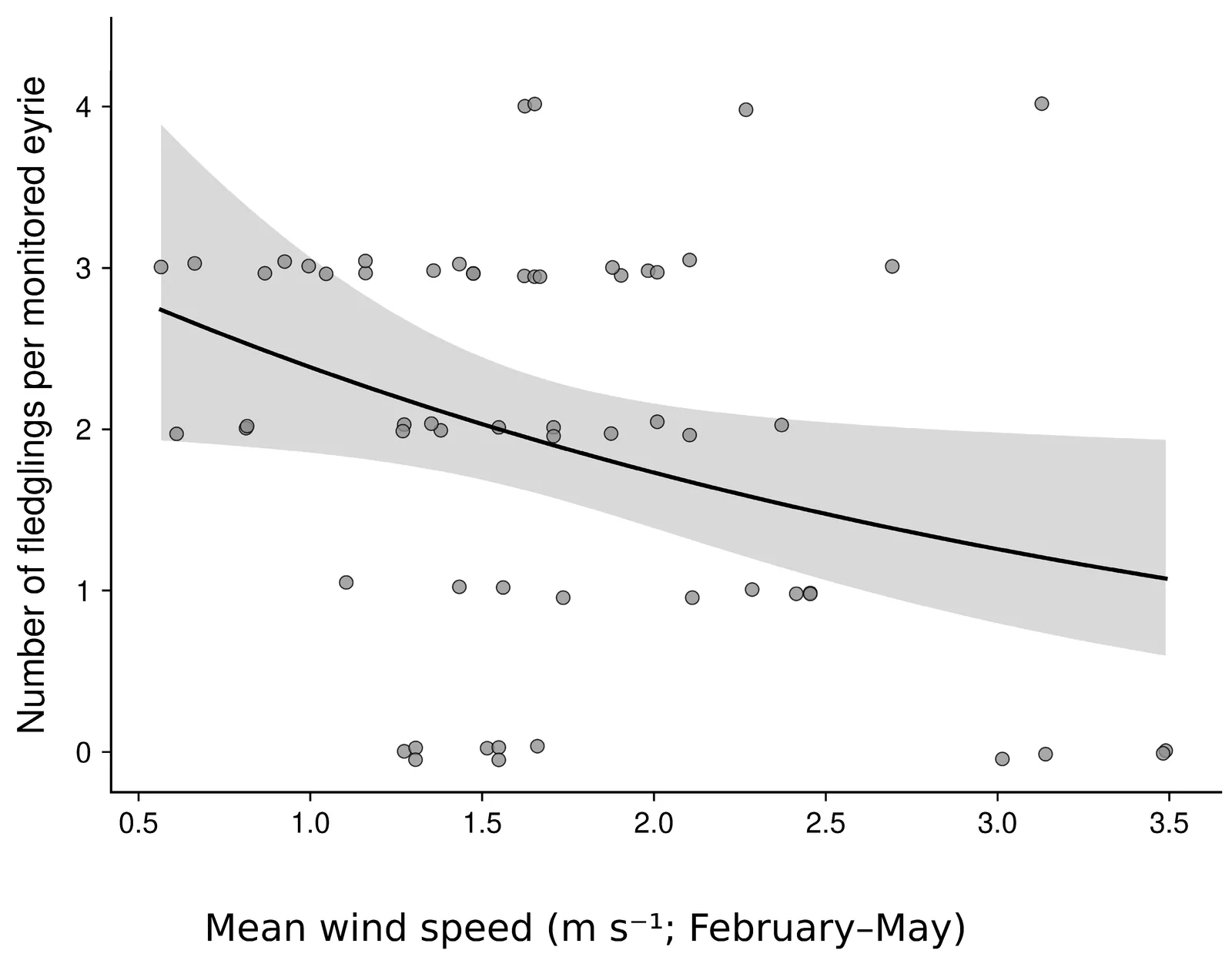
Relationship between February–May mean wind speed and breeding productivity predicted by the best ranked additive Poisson model. The line is the expected number of fledglings with eyrie cliff–wind alignment (CosDiff) held at its mean; shading is the model-based 95% confidence interval. Points are observed eyrie-level counts with slight vertical jitter for visibility.

**Figure 4:**
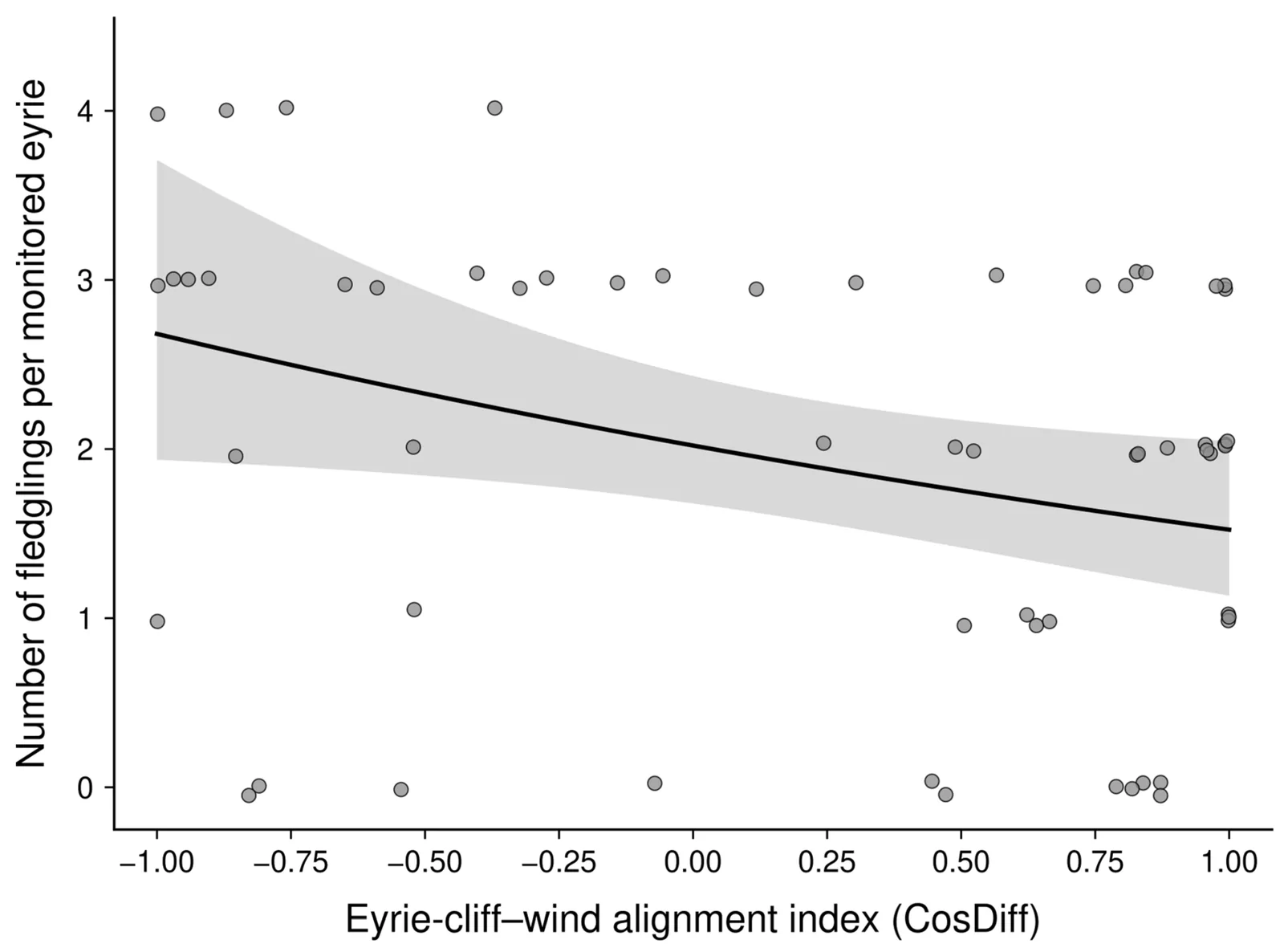
Relationship between eyrie cliff–wind alignment (CosDiff) and breeding productivity predicted by the best ranked additive Poisson model. Seasonal mean wind speed is held at its mean; shading is the model-based 95% confidence interval. Higher CosDiff indicates more direct alignment with incoming prevailing wind.

In M6, a one standard deviation increase in seasonal mean wind speed (0.678 m s^−1^) was associated with a 19.5% decrease in expected fledgling production (IRR = 0.805, 95% CI = 0.660–0.982, *p* = 0.032; Table 4; Figure 3). A one standard deviation increase in CosDiff, representing more direct alignment of the eyrie cliff face with incoming prevailing wind, was associated with an 18.7% decrease (IRR = 0.813, 95% CI = 0.677–0.976, *p* = 0.026; Table 4; Figure 4). Adding alignment to the wind-only model improved fit (likelihood-ratio χ^2^ = 4.855, df = 1, *p* = 0.028).

### 3.3. Climatic Interactions and Distributional Sensitivity

The seasonal climatic model containing mean wind speed, cumulative precipitation, and minimum air temperature received less support than M6 (Table S2). Within that model, wind remained negative, whereas precipitation and minimum air temperature were unsupported (Table S2). In the core global additive model, all estimates retained their expected directions, but the wind confidence interval narrowly included no effect, and pair type, precipitation, minimum air temperature, and alignment were also individually uncertain (Table S2).

The wind by precipitation interaction did not improve the seasonal climatic model, and the wind by alignment interaction did not improve M6 (Table S2). The corresponding COM–Poisson tests were also unsupported (Table S2).

The COM–Poisson M6 model closely reproduced the Poisson estimates for wind and alignment (Table 4). This agreement indicates that the focal associations were not artefacts of the Poisson variance assumption.

### 3.4. Monthly Temporal Sensitivity

Wind-only estimates were similar across February, March, and April (Table S3). Under Poisson regression, the confidence intervals included the null value, whereas the corresponding COM–Poisson estimates provided support for a negative wind association (Table S3). No month showed a distinctly stronger wind association, and none of the six wind by weather interactions were supported after Holm correction (Table S3).

### 3.5. Diagnostics, Influence, and Topographic Sensitivity

The focal Poisson M6 model showed no evidence of residual non-uniformity, a dispersion anomaly, zero inflation, outliers, or residual spatial autocorrelation (Table S4). Corresponding COM–Poisson diagnostics were also satisfactory (Table S4).

Across all 59 leave-one-eyrie-out refits, the wind and alignment coefficients remained negative under both distributions (Table S4). Thus, the direction of these effects was not driven by a single eyrie, although statistical support varied in some refits.

Topographic measurements were complete for 39 eyries and missing for 20. Mean productivity was similar between groups, but seasonal mean wind speed was higher in the complete group (Table S5). Within the 39-eyrie subset, the wind-only model ranked first; adding elevation, eyrie height, or both jointly did not improve model fit (Table S5). Because topographic completeness was associated with wind exposure and the subset was smaller, these results are sensitivity evidence rather than definitive tests of topographic effects.

### 3.6. Eyrie Cliff Aspects and Prevailing Wind

**Figure 5:**
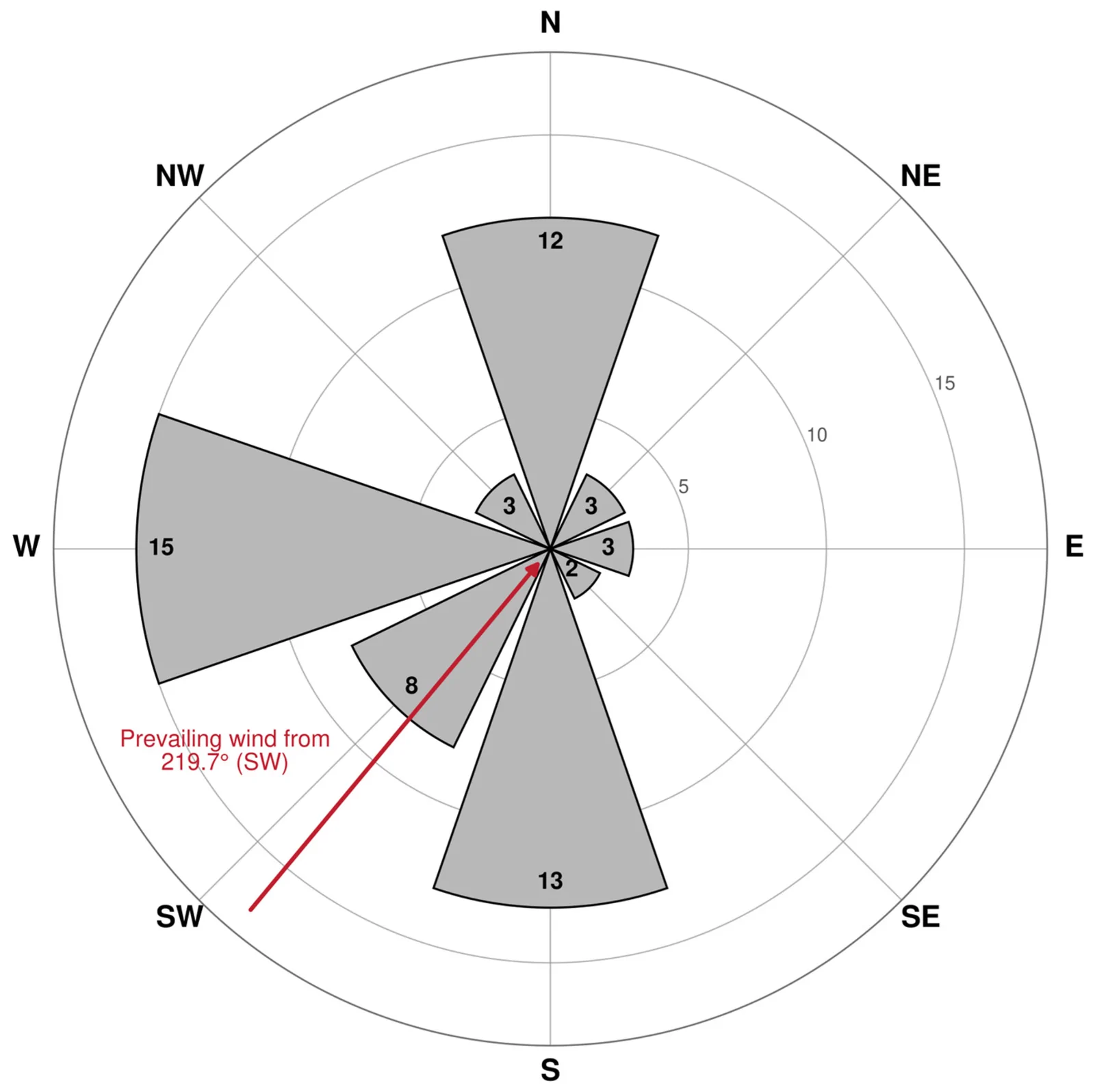
Distribution of eyrie cliff aspects among 59 monitored eyries. Numbers inside bars are eyrie counts. The red inward arrow indicates the meteorological direction from which the circular mean prevailing wind arrived (219.7°, southwest).

Eyrie cliff aspects were non-uniform across the eight sectors (Table S6; Figure 5). West was the most frequent sector, followed by south and north (Figure 5). After Holm correction of sector-level residual tests, west was the only sector occurring more frequently than expected under the equal frequency distribution. Because directional availability of suitable cliffs was not quantified, this finding indicates uneven use rather than directional preference.

Prevailing winds were strongly concentrated in the southwest sector, followed by south (Figure 5; Table S6). This concentration reflects the spatially coherent ERA5-Land resultant wind vectors at the sampled eyries during 2026, not an arithmetic average of directional bearings; it should therefore not be generalized beyond the sampled locations and breeding season.

The relative eyrie cliff–wind analysis showed evidence of non-uniformity before multiple testing correction (Table S6). However, neither the Rayleigh nor Kuiper test remained significant after Holm correction across the five related tests. Most eyries were oblique to the local prevailing wind direction, whereas fewer were directly aligned or approximately opposite (Table S6). The three-class distribution did not differ from its angular null expectation, and oblique aspects were not enriched relative to their corresponding null expectation (Table S6). The uncorrected signal for the narrower band centred on 45° also did not remain significant after Holm correction. Overall, the data did not support a fixed approximately 45° optimum or a general excess of oblique aspects (Table S6).

## 4. Discussion

### 4.1. Seasonal Wind Exposure and Breeding Productivity

Seasonal mean wind speed and eyrie cliff alignment with incoming prevailing wind were the two supported correlates of Barbary falcon productivity in the best ranked model. A one standard deviation increase in either predictor corresponded to an approximately 19% reduction in expected fledgling production. Both associations were similar under COM–Poisson modelling and retained a negative direction in every leave-one-eyrie-out refit. Month-specific analyses showed comparable wind estimates across February, March, and April rather than identifying one month as a uniquely influential period. All temporal interpretations remain exploratory because the data represent one breeding season and do not establish causation or a wind speed threshold.

Several non-exclusive mechanisms may underlie this association. Wind can affect flight performance and access to cliff nests; for example, strong winds substantially reduced successful landing attempts in cliff-nesting birds [36]. Although this evidence was obtained from seabirds rather than raptors, it provides a plausible aerodynamic mechanism relevant to cliff eyries. In Gyrfalcons, greater horizontal exposure at nesting sites was associated with increased adult nest attendance and reduced productivity, indicating that structural shelter can influence both adult behaviour and reproductive performance [3]. Wind may also increase exposure to adverse weather by driving precipitation into otherwise sheltered eyries [2].

Mean productivity in the present study (1.97 fledglings per monitored eyrie) was higher than the estimate of 1.33 fledglings per pair reported in Saudi Arabia in 2015 [16]. It was within the range of 1.62–2.53 fledglings per territorial pair summarized for Barbary falcon populations in the Canary Islands, but lower than the 3.43 ± 0.79 fledglings per territorial pair reported from seven breeding attempts monitored across four eyries in southwestern Iran [37,38]. Differences in productivity definitions and denominators, sample sizes, monitoring protocols, breeding seasons, and environmental conditions preclude formal temporal or geographic comparisons; these estimates are presented only to provide demographic context.

### 4.2. Precipitation, Temperature, and Interaction Hypotheses

Precipitation and minimum air temperature received little support in the seasonal or month-specific models. The seasonal wind by precipitation interaction was unsupported. Across February, March, and April, neither the wind by precipitation nor the wind by minimum air temperature interaction was supported after Holm correction. Thus, the present analyses did not detect a compound weather relationship, although their exploratory scope and single-season design limit inference.

Nevertheless, precipitation and low temperature remain biologically plausible influences on falcon reproduction. Experimental work on American kestrels (*Falco sparverius*) showed that rainfall increased metabolic rate, while lower ambient temperatures were also associated with greater metabolic demands [39]. In Peregrine falcons, nestling mortality was positively correlated with precipitation recorded during prolonged storms [40], while experimental protection from rainfall increased nestling survival and confirmed that heavy rainfall can directly cause mortality [5]. In South Greenland, breeding success was negatively associated with the number of days characterized by extreme weather and extremely low temperatures [6]. These studies were conducted in markedly different climatic environments and primarily evaluated intense events or numbers of extreme weather days. By contrast, the seasonal predictors used in the present study summarized cumulative precipitation and minimum air temperature conditions across February–May in a predominantly warm and arid environment. Such temporal aggregation may obscure short but biologically important weather events.

### 4.3. Topography and Directly Confirmed Nest Attributes

Elevation and eyrie height did not improve the wind-based Poisson models fitted to the 39 eyries with complete topographic measurements. These variables therefore provided no detectable incremental explanatory value within that subset. However, topographic completeness was associated with wind exposure: the 39 complete sites were windier on average than the 20 sites without measurements, although productivity did not differ. Together with the smaller subset and the accessibility-based reference used for eyrie height, this pattern limits representativeness and prevents interpreting the null topographic additions as evidence that topography is unimportant across the Saudi range.

This cautious interpretation is consistent with evidence that breeding site occupancy and reproductive performance do not necessarily respond to the same habitat characteristics. In Tenerife, Barbary falcons selected taller coastal cliffs and sites farther from roads and buildings, but the measured habitat characteristics were not correlated with productivity [41]. Conversely, among 67 Peregrine falcon nesting cliffs monitored in central West Greenland from 1972 to 1999, consistently occupied cliffs had higher mean productivity and lower temporal variation in productivity than inconsistently occupied cliffs. High-quality habitat was characterized by taller cliffs, greater local elevation gain, better weather protection at the eyrie ledge, and greater isolation from neighbouring cliffs [19]. Studies of Bonelli’s eagle nest/cliff selection and insular cliff-nesting raptor communities further demonstrate that cliff geometry, landscape context, and human disturbance can influence breeding site occupancy [7,20]. The present subset analysis therefore indicates an absence of a detectable independent topographic association with annual fledgling productivity rather than a general absence of biological importance.

Nest form, nest substrate or lining, clutch size, and nest entrance aspect were reported descriptively only for the 15 nests in which eggs and structural characteristics were directly confirmed. These nest-level measurements were distinct from the eyrie cliff aspect, which described the orientation of the occupied cliff at all 59 monitored eyries and was analysed separately. Cavities and sand were the most frequently recorded categories within the directly verified subset. The predominance of cavities was consistent with the exclusive use of cliff cavities reported at four Barbary falcon eyries in southwestern Iran [38], while cavity and ledge nesting was documented among nine monitored nests in La Palma [37]. However, the small and non-randomly observed subset does not permit population-level estimates or reliable comparisons of productivity among nest structure categories.

### 4.4. Eyrie Cliff Aspect in Relation to Prevailing Wind

The distribution of eyrie cliff aspects was non-uniform, but only the western sector occurred more frequently than the equal frequency expectation after correction for multiple comparisons. This finding indicates uneven directional use rather than directional selection because the availability and quality of suitable cliff faces in each aspect class was not quantified [33]. Because eyrie structure and shelter may also influence breeding site quality [2,19], infrequent aspects should not be interpreted as evidence of behavioural avoidance.

The continuous CosDiff model indicated lower productivity where eyrie cliff faces were more directly aligned with the direction from which the prevailing wind arrived. CosDiff quantifies the angular correspondence between cliff face aspect and prevailing wind direction; it does not directly measure airflow, turbulence, or shelter within the eyrie. Because eyrie cliff aspect was represented by eight compass sector midpoints rather than exact bearings, CosDiff should be interpreted as an approximate alignment index. More direct alignment could plausibly increase wind exposure at the cliff face, but this mechanism was not measured and requires cliff-scale validation. This directional association does not imply a universal preferred compass sector: the categorical and circular tests did not support a fixed directional optimum, including an approximately 45° orientation rule, after Holm correction. Local wind direction and cliff shelter should therefore be evaluated together rather than using aspect alone.

### 4.5. Study Limitations and Research Priorities

Inference is limited by the single 2026 breeding season, the observational design, and the concentration of monitored eyries in two broad spatial clusters. The sample was not spatially continuous or probability-based across the species’ Saudi range and therefore may not represent conditions in unmonitored regions. Consequently, this study cannot establish causality or determine whether the identified associations of wind conditions and eyrie cliff alignment with fledgling productivity persist across years.

Productivity may also have been influenced by unmeasured factors, including prey availability, disturbance, and adult experience. ERA5-Land’s approximately 9 km resolution cannot resolve cliff-scale airflow or shelter, and short-term behavioural responses to UAV surveys were not quantified. Differences in visit timing were partly mitigated by weekly local monitoring and cross-checking of final fledgling counts.

Topographic measurements were available for 39 eyries, directly confirmed nest attributes for 15 nests, and only 15 pairs were release-supported. These limitations, together with the relatively broad analytical scope for 59 eyries, reduce the power of some analyses and warrant cautious interpretation of the monthly, topographic, and directional results.

Future studies should combine multi-year monitoring with direct cliff-level wind measurements, complete topographic coverage, breeding stage-specific weather metrics, and systematic assessments of prey availability and disturbance.

## 5. Conclusions

In this single-season study, higher seasonal mean wind speed and more direct eyrie cliff alignment with incoming prevailing wind were associated with lower fledgling productivity in Barbary falcon eyries. These preliminary associations may provide useful considerations for future release site evaluation but should be interpreted within a broader assessment that includes demographic context, prey availability, threats, the presence of a suitable natural eyrie, and monitoring feasibility [42]. Wind exposure and eyrie cliff aspect should not be used independently to select or exclude release sites, and multi-year studies incorporating cliff-scale measurements are needed to determine whether these associations persist and have practical value for release site evaluation.

The absence of a detectable pair-type effect should not be interpreted as evidence of equivalence between naturally formed and release-supported pairs. Where releases involve territories occupied by a resident unpaired adult, continued monitoring of survival, settlement, pair persistence, eyrie use, and fledgling production is recommended.

## Figures and Tables

**Table 1:** Descriptive statistics for breeding productivity and environmental and topographic variables. Weather summaries represent February–May 2026; mean wind speed is the arithmetic mean of daily 10 m wind speed magnitudes. Topographic variables were available for 39 eyries; all other variables were available for 59 eyries.

Variable	Unit	N	Mean ± SD	Median [IQR]	Range
Breeding productivity	fledgling eyrie^−1^	59	1.97 ± 1.25	2.00 [1.00–3.00]	0.00–4.00
Seasonal mean wind speed	m s^−1^	59	1.71 ± 0.68	1.62 [1.29–2.06]	0.57–3.49
Seasonal precipitation	mm	59	115.67 ± 73.27	127.47 [34.98–161.48]	16.56–314.43
Seasonal minimum air temperature	°C	59	7.31 ± 2.44	7.56 [5.49–8.74]	1.79–14.41
Eyrie cliff–wind alignment index (CosDiff)	unitless	59	0.22 ± 0.73	0.51 [−0.52–0.86]	−1.00–1.00
Eyrie elevation	m a.s.l.	39	1729.41 ± 536.40	1755.00 [1330.85–2146.20]	746.20–2681.00
Eyrie height on cliff	m	39	167.44 ± 117.18	120.00 [75.00–250.00]	20.00–500.00

**Table 2:** Directly confirmed nest attributes for the 15 nests in which eggs and structural characteristics were observed. These variables were summarized descriptively and were not entered into productivity models.

Attribute	Category	N	%
Nest form	Cavity	11	73.3
	Crack	4	26.7
Substrate or lining	Sand	14	93.3
	Confirmed old raptor nest	1	6.7
Clutch size	1 egg	1	6.7
	2 eggs	2	13.3
	3 eggs	7	46.7
	4 eggs	5	33.3
Nest entrance aspect	N	5	33.3
	NE	1	6.7
	E	2	13.3
	S	5	33.3
	SW	1	6.7
	W	1	6.7

**Table 3:** Complete Poisson candidate model ranking for fledgling productivity (N = 59). All weather variables summarize February–May 2026. K is the number of estimated regression parameters, including the intercept. Models with ΔAICc ≤ 2 were considered similarly supported and are shown in bold. Corresponding COM–Poisson rankings are reported in Table S2.

Rank	Model	Ecological Association Represented	K	logLik	AICc	ΔAICc	Weight
1	M6	Mean wind + eyrie cliff–wind alignment	3	−93.586	193.609	0.000	0.409
2	M7	Mean wind × eyrie cliff–wind alignment	4	−93.073	194.887	1.278	0.216
3	M5	Eyrie cliff–wind alignment	2	−95.999	196.212	2.603	0.111
4	M2	Mean wind	2	−96.014	196.242	2.633	0.110
5	M0	Null	1	−97.621	197.312	3.704	0.064
6	M3	Seasonal climate: wind + rain + minimum air temperature	4	−94.801	198.342	4.733	0.038
7	M1	Pair type: naturally formed vs. release-supported	2	−97.616	199.445	5.837	0.022
8	M8	Core global additive model	6	−93.085	199.784	6.176	0.019
9	M4	Mean wind × rain + minimum air temperature	5	−94.786	200.705	7.096	0.012

**Table 4:** Standardized seasonal mean wind speed and eyrie cliff–wind alignment (CosDiff) estimates from the focal February–May model under Poisson and COM–Poisson distributions. IRRs represent a one standard deviation increase in the predictor.

Distribution	Model	Predictor	Estimate	SE	IRR	95% CI	*p*
Poisson	M6: wind + CosDiff	Mean wind speed	−0.217	0.101	0.805	0.660–0.982	0.032
Poisson	M6: wind + CosDiff	Eyrie cliff–wind alignment (CosDiff)	−0.207	0.093	0.813	0.677–0.976	0.026
COM–Poisson	M6: wind + CosDiff	Mean wind speed	−0.212	0.088	0.809	0.680–0.962	0.017
COM–Poisson	M6: wind + CosDiff	Eyrie cliff–wind alignment (CosDiff)	−0.205	0.082	0.815	0.694–0.956	0.012

Notes: For Poisson models, adding alignment to wind yielded likelihood ratio *p* = 0.028, whereas adding the wind × alignment interaction to M6 yielded *p* = 0.311; the corresponding COM–Poisson *p* values were 0.015 and 0.247, respectively.

## Data Availability

The data supporting the findings of this study are available from the corresponding author upon reasonable request and subject to approval by the National Centre for Falcons. The data are not publicly available because they include sensitive breeding site locations of a protected raptor and are subject to institutional ownership and conservation restrictions. Any data sharing will exclude precise eyrie coordinates or provide spatially generalized locations, as appropriate, to protect active breeding sites.

## Supplementary Materials

The following supporting information can be downloaded at: https://www.mdpi.com/article/10.3390/life16091482/s1: Table S1: Pearson correlation matrix for February–May 2026 climatic summaries (N = 59); Table S2: Complete seasonal candidate-model definitions and corrected model comparisons; Table S3: February–April temporal sensitivity analyses; Table S4: Diagnostics, influence screening, and leave-one-eyrie-out sensitivity; Table S5: Topographic missingness and Poisson sensitivity analyses; Table S6: Directional distributions and circular tests.
