# Supplementary material for: Environmental Variables and Eyrie Site Characteristics Associated with Breeding Productivity of the Barbary Falcon (Falco peregrinus pelegrinoides): Potential Implications for Release Site Evaluation
Source: Life (Basel). 2026 Sep 5;16(9):1482. doi: 10.3390/life16091482 (PMC13608579; doi:10.3390/life16091482)
Supplement: Supplementary file 1 [file life-16-01482-s001.zip › life-4521383-supplementary.pdf]

Supplementary Materials

Table S1. Pearson correlation matrix for February–May 2026 climatic summaries (N = 59).

Variable	Seasonal mean wind speed	Seasonal maximum wind speed	Seasonal precipitation	Seasonal minimum air temperature	Seasonal mean air temperature	Seasonal maximum air temperature
Seasonal mean wind speed	1.000	0.915	-0.679	-0.687	0.228	0.470
Seasonal maximum wind speed	0.915	1.000	-0.571	-0.657	0.063	0.262
Seasonal precipitation	-0.679	-0.571	1.000	0.527	-0.527	-0.703
Seasonal minimum air temperature	-0.687	-0.657	0.527	1.000	0.191	-0.141
Seasonal mean air temperature	0.228	0.063	-0.527	0.191	1.000	0.890
Seasonal maximum air temperature	0.470	0.262	-0.703	-0.141	0.890	1.000

Note: The productivity candidate models retained mean wind speed, cumulative precipitation, and minimum air temperature. Variables with $|r| \geq 0.70$ within the same climatic domain were treated as redundant. All wind-containing models use the daily-magnitude-based mean-speed predictor described in the main-text Methods.

Table S2. Complete seasonal candidate-model definitions and corrected model comparisons.

Panel A. *Candidate structures.*

Model	Short label	Structure
M0	Null	Intercept only
M1	Pair type: naturally formed vs release-supported	Naturally formed vs release-supported pair
M2	Mean wind	Seasonal mean wind speed
M3	Seasonal climate: wind + rain + minimum air temperature	Mean wind + rain + minimum air temperature
M4	Mean wind × rain + minimum air temperature	M3 + mean wind × rain
M5	Eyrie cliff–wind alignment	Eyrie cliff–wind alignment (CosDiff)
M6	Mean wind + eyrie cliff–wind alignment	Mean wind + CosDiff
M7	Mean wind × eyrie cliff–wind alignment	M6 + mean wind × CosDiff
M8	Core global additive model	Pair type + mean wind + rain + minimum air temperature + CosDiff

Table S2. Continued.

Panel B. *Poisson candidate-model ranking.*

Rank	Model	K	logLik	AICc	ΔAICc	Akaike weight
1	M6	3	-93.586	193.609	0.000	0.409
2	M7	4	-93.073	194.887	1.278	0.216
3	M5	2	-95.999	196.212	2.603	0.111
4	M2	2	-96.014	196.242	2.633	0.110

Rank	Model	K	logLik	AICc	ΔAICc	Akaike weight
5	M0	1	-97.621	197.312	3.704	0.064
6	M3	4	-94.801	198.342	4.733	0.038
7	M1	2	-97.616	199.445	5.837	0.022
8	M8	6	-93.085	199.784	6.176	0.019
9	M4	5	-94.786	200.705	7.096	0.012

Table S2. Continued.

Panel C. *COM–Poisson candidate-model ranking.*

Rank	Model	K	logLik	AICc	ΔAICc	Akaike weight
1	M6	4	-92.613	193.966	0.000	0.463
2	M7	5	-91.942	195.017	1.051	0.274
3	M5	3	-95.534	197.505	3.539	0.079
4	M2	3	-95.580	197.596	3.630	0.075
5	M0	2	-97.416	199.046	5.080	0.037
6	M3	5	-94.146	199.424	5.459	0.030
7	M8	7	-91.960	200.116	6.150	0.021
8	M1	3	-97.410	201.256	7.290	0.012
9	M4	6	-94.121	201.857	7.891	0.009

Table S2. Continued.

Panel D. *Nested likelihood-ratio tests.*

Distribution	Comparison	χ^2	df	P
Poisson	M6 versus M2: add alignment to wind	4.855	1	0.028
Poisson	M7 versus M6: add wind × alignment	1.026	1	0.311
Poisson	M4 versus M3: add wind × precipitation	0.029	1	0.865
COM–Poisson	M6 versus M2: add alignment to wind	5.934	1	0.015
COM–Poisson	M7 versus M6: add wind × alignment	1.340	1	0.247
COM–Poisson	M4 versus M3: add wind × precipitation	0.051	1	0.822

Table S2. Continued.

Panel E. *Selected Poisson coefficient estimates cited in the main-text Results.*

Model	Predictor	Estimate	SE	IRR	95% CI	P
M1	Pair type: release-supported vs naturally formed	-0.022	0.214	0.978	0.642–1.489	0.917

Model	Predictor	Estimate	SE	IRR	95% CI	P
M3	Mean wind speed	-0.388	0.183	0.678	0.474–0.971	0.034
M3	Seasonal precipitation	-0.179	0.136	0.836	0.641–1.091	0.187
M3	Minimum air temperature	-0.098	0.140	0.907	0.689–1.193	0.483
M8	Pair type: release-supported vs naturally formed	0.032	0.238	1.033	0.647–1.648	0.892
M8	Mean wind speed	-0.362	0.186	0.696	0.484–1.002	0.051
M8	Seasonal precipitation	-0.109	0.143	0.897	0.678–1.187	0.448
M8	Minimum air temperature	-0.080	0.143	0.923	0.697–1.221	0.574
M8	Eyrie cliff–wind alignment (CosDiff)	-0.180	0.097	0.835	0.690–1.011	0.064

Note: Pair type compares release-supported with naturally formed pairs. Continuous predictors were standardized; IRRs and 95% CIs represent a one-standard-deviation increase for continuous predictors.

Note: All continuous predictors were standardized using full-sample means and standard deviations. The candidate set was exploratory and biologically structured after correlation screening; it was not preregistered.

Table S3. February–April temporal sensitivity analyses.

Panel A. *Month-specific climatic descriptive statistics.*

Month	Variable	N	Mean	SD	Median	IQR	Range
February	Mean wind speed (m s^{-1})	59	1.501	0.747	1.278	1.032–1.767	0.500–4.177
February	Precipitation (mm)	59	2.019	1.758	2.033	0.037–3.148	0.028–6.554
February	Minimum air temperature (°C)	59	8.256	2.057	8.493	7.209–9.323	3.790–14.412
March	Mean wind speed (m s^{-1})	59	2.004	0.693	1.916	1.634–2.352	0.510–3.816
March	Precipitation (mm)	59	71.054	46.989	72.660	23.339–92.545	12.541–185.892
March	Minimum air temperature (°C)	59	8.465	3.401	8.373	6.226–10.163	1.786–16.874
April	Mean wind speed (m s^{-1})	59	1.797	0.659	1.783	1.347–2.183	0.493–3.391
April	Precipitation (mm)	59	33.306	24.315	23.965	13.045–49.678	3.502–105.209
April	Minimum air temperature (°C)	59	12.355	2.574	12.847	10.547–13.718	6.468–19.169

Table S3. Continued.

Panel B. *Poisson candidate-model rankings.*

Month	Model	K	AICc	ΔAICc	Akaike weight
February	Wind only	2	195.438	0.000	0.309

Month	Model	K	AICc	ΔAICc	Akaike weight
February	Additive weather	4	195.526	0.088	0.295
February	Null	1	197.312	1.874	0.121
February	Wind × precipitation	5	197.791	2.352	0.095
February	Wind × minimum air temperature	5	197.876	2.438	0.091
February	Precipitation only	2	199.205	3.767	0.047
February	Minimum air temperature only	2	199.434	3.995	0.042
March	Wind only	2	195.806	0.000	0.397
March	Null	1	197.312	1.507	0.187
March	Additive weather	4	197.903	2.097	0.139
March	Minimum air temperature only	2	198.597	2.791	0.098
March	Precipitation only	2	199.454	3.648	0.064
March	Wind × precipitation	5	199.539	3.734	0.061
March	Wind × minimum air temperature	5	199.844	4.038	0.053
April	Wind only	2	195.595	0.000	0.416
April	Null	1	197.312	1.718	0.176
April	Additive weather	4	197.448	1.853	0.165
April	Precipitation only	2	199.073	3.479	0.073
April	Minimum air temperature only	2	199.430	3.835	0.061
April	Wind × minimum air temperature	5	199.504	3.909	0.059
April	Wind × precipitation	5	199.820	4.225	0.050

Table S3. Continued.

Panel C. *COM–Poisson candidate-model rankings.*

Month	Model	K	AICc	ΔAICc	Akaike weight
February	Additive weather	5	195.869	0.000	0.382
February	Wind only	3	196.690	0.821	0.253
February	Wind × precipitation	6	198.225	2.356	0.118
February	Wind × minimum air temperature	6	198.301	2.431	0.113
February	Null	2	199.046	3.177	0.078
February	Precipitation only	3	200.985	5.116	0.030
February	Minimum air temperature only	3	201.242	5.373	0.026
March	Wind only	3	197.071	0.000	0.400

Month	Model	K	AICc	ΔAICc	Akaike weight
March	Additive weather	5	198.727	1.656	0.175
March	Null	2	199.046	1.975	0.149
March	Wind × precipitation	6	200.289	3.218	0.080
March	Minimum air temperature only	3	200.291	3.220	0.080
March	Wind × minimum air temperature	6	200.656	3.586	0.067
March	Precipitation only	3	201.265	4.194	0.049
April	Wind only	3	196.799	0.000	0.425
April	Additive weather	5	198.281	1.482	0.203
April	Null	2	199.046	2.247	0.138
April	Wind × minimum air temperature	6	200.373	3.574	0.071
April	Wind × precipitation	6	200.737	3.938	0.059
April	Precipitation only	3	200.835	4.036	0.057
April	Minimum air temperature only	3	201.239	4.440	0.046

Table S3. Continued.

Panel D. *Wind-only coefficient estimates.*

Month	Distribution	Estimate	SE	IRR	95% CI	P
February	Poisson	-0.208	0.110	0.812	0.655–1.007	0.057
March	Poisson	-0.183	0.096	0.833	0.690–1.006	0.058
April	Poisson	-0.186	0.095	0.830	0.689–1.001	0.051
February	COM–Poisson	-0.205	0.100	0.815	0.669–0.992	0.041
March	COM–Poisson	-0.181	0.088	0.834	0.702–0.991	0.040
April	COM–Poisson	-0.185	0.087	0.831	0.701–0.985	0.033

Table S3. Continued.

Panel E. *Monthly Poisson interaction likelihood-ratio tests.*

Month	Interaction	χ^2	df	Unadjusted P	Holm-adjusted P
February	Wind × precipitation	0.127	1	0.722	1.000
February	Wind × minimum air temperature	0.041	1	0.840	1.000
March	Wind × precipitation	0.755	1	0.385	1.000
March	Wind × minimum air temperature	0.450	1	0.502	1.000
April	Wind × precipitation	0.019	1	0.889	1.000
April	Wind × minimum air temperature	0.335	1	0.562	1.000

Note: Holm adjustment was applied jointly to the six interaction tests. The monthly analyses were exploratory and were used to compare the direction and magnitude of associations across February, March, and April.

Table S4. Diagnostics, influence screening, and leave-one-eyrie-out sensitivity.

Panel A. *DHARMa residual diagnostics.*

Model	Uniformity P	Dispersion P	Zero-inflation P	Outlier P
Poisson M6	0.191	0.071	0.652	1.000
COM–Poisson M6	0.290	0.586	0.148	1.000

Table S4. Continued.

Panel B. *Residual spatial-autocorrelation test.*

Model	Observed Moran's I	Expected Moran's I	P
Poisson M6	0.068	-0.017	0.123

Table S4. Continued.

Panel C. *Eyries flagged in the focal Poisson M6 model.*

Row	Site ID	Cook's distance	Leverage	Std. Pearson residual	Cook flag	Leverage flag	Residual flag
2	H3	0.1509	0.1003	2.015	Yes	No	Yes
21	M13	0.0807	0.1281	-1.284	Yes	Yes	No
31	B6	0.0874	0.0768	-1.775	Yes	No	No
48	J3	0.0229	0.2261	-0.485	No	Yes	No

Table S4. Continued.

Panel D. *Full leave-one-eyrie-out coefficient stability.*

Distribution	Term	Deletions	Estimate range	IRR range	P-value range	Negative in all	P < 0.05 in all
Poisson	Mean wind speed	59	-0.276 to -0.175	0.759–0.839	0.011–0.095	Yes	No
Poisson	Eyrie cliff–wind alignment	59	-0.243 to -0.185	0.784–0.831	0.010–0.053	Yes	No
COM–Poisson	Mean wind speed	59	-0.267 to -0.169	0.766–0.844	0.004–0.059	Yes	No
COM–Poisson	Eyrie cliff–wind alignment	59	-0.241 to -0.183	0.786–0.832	0.002–0.028	Yes	Yes

Note: Residual diagnostics used 5000 simulations. Influence thresholds were $4/n = 0.0678$ for Cook's distance, $2p/n = 0.1017$ for leverage ($p = 3$), and |standardized Pearson residual| > 2. All 59 deletion refits converged for each distribution.

Table S5. Topographic missingness and Poisson sensitivity analyses.

Panel A. *Descriptive comparison of eyries with and without complete topographic measurements.*

Group	Variable	N	Mean	SD	Median	IQR	Range
Missing (n = 20)	Seasonal mean wind speed	20	1.441	0.373	1.406	1.245–1.672	0.816–2.112
Complete (n = 39)	Seasonal mean wind speed	39	1.850	0.758	1.668	1.393–2.329	0.566–3.489

Group	Variable	N	Mean	SD	Median	IQR	Range
Missing (n = 20)	Fledglings 2026	20	1.900	1.252	2.000	0.750–3.000	0.000–3.000
Complete (n = 39)	Fledglings 2026	39	2.000	1.257	2.000	1.000–3.000	0.000–4.000

Table S5. Continued.

Panel B. *Missingness-group comparisons.*

Variable	Welch t	Welch P	Mann–Whitney P
Seasonal mean wind speed	-2.776	0.007	0.034
Fledglings 2026	-0.290	0.773	0.855

Table S5. Continued.

Panel C. *Poisson AICc ranking for the 39 complete eyries.*

Rank	Model	K	AICc	ΔAICc	Akaike weight
1	Wind only	2	129.795	0.000	0.424
2	Wind + elevation	3	130.415	0.620	0.311
3	Wind + eyrie height	3	131.731	1.936	0.161
4	Wind + elevation + eyrie height	4	132.589	2.794	0.105

Table S5. Continued.

Panel D. *Likelihood-ratio tests for adding topographic variables to wind.*

Comparison	χ^2	df	P
Add elevation	1.732	1	0.188
Add eyrie height	0.417	1	0.519
Add elevation and eyrie height	2.050	2	0.359

Note: Elevation and eyrie height were standardized. Eyrie height was measured from the nearest point accessible without climbing equipment and should not be interpreted as total cliff height.

Table S6. Directional distributions and circular tests.

Panel A. *Eyrie cliff aspects.*

Eyrie cliff aspect	Central bearing (°)	N	%
N	0	12	20.3
NE	45	3	5.1
E	90	3	5.1
SE	135	2	3.4
S	180	13	22.0
SW	225	8	13.6
W	270	15	25.4
NW	315	3	5.1

Table S6. Continued.

Panel B. *February–May prevailing-wind sectors.*

Wind direction	Central bearing (°)	N	%
N	0	0	0.0
NE	45	0	0.0
E	90	0	0.0
SE	135	1	1.7
S	180	11	18.6
SW	225	42	71.2
W	270	3	5.1
NW	315	2	3.4

Table S6. Continued.

Panel C. *Directional tests.*

Target	Test	Statistic	Unadjusted P	Interpretation
Eyrie cliff sectors	Equal-frequency chi-square	$\chi^2 = 26.831$; df = 7	<0.001	West was the only sector above expectation after Holm correction
Prevailing wind	Rayleigh	Z = 44.743; r = 0.871	<0.001	Strong southwesterly concentration; circular mean = 219.75°
Relative eyrie cliff–wind angle	Rayleigh	Z = 3.648; r = 0.249	0.026	Weak unimodal signal; not significant after Holm correction
Relative eyrie cliff–wind angle	Kuiper (99,999 simulations)	V = 0.233	0.030	General departure signal; not significant after Holm correction
Eyrie cliff orientation relative to wind	Three-class chi-square	$\chi^2 = 3.780$; df = 2	0.151	No departure from angular null expectation
Oblique eyrie cliff aspects	Exact binomial vs 0.75	42/59 (71.2%)	0.798	No enrichment of oblique aspects
Eyrie cliff aspects approximately 45° from wind	Exact binomial vs 0.25	21/59 (35.6%)	0.046	Uncorrected signal; not significant after Holm correction

Note: The eyrie-cliff–wind alignment index (CosDiff) used the central bearings of the eight recorded eyrie cliff aspect sectors and the local seasonal prevailing-wind bearing. The five relative-angle tests were Holm-corrected as one family. The corresponding Holm-adjusted P values for the five relative-angle tests, in the order shown, were 0.130, 0.130, 0.302, 0.798, and 0.138, respectively.
